# Psychological impact of pediatric cancer : a cross-sectional study among thirty parents

**DOI:** 10.1192/j.eurpsy.2022.1692

**Published:** 2022-09-01

**Authors:** A. Zouari, W. Homri, A. Mokrani, K. Meddeb, A. Mezelini, R. Labbane

**Affiliations:** 1 Razi Hospital, Psychiatry C, mannouba, Tunisia; 2 salah azaeiz hospital, Medical Oncology, tunis, Tunisia

**Keywords:** cancer, PTSD, Child, Depression

## Abstract

**Introduction:**

The diagnosis of pediatric cancer is a traumatic event that is considered to be one of the most adverse situations the child and his family can experience. The psychological impact of this diagnosis on the parents has triggered a great scientific attention in these recent years.

**Objectives:**

Estimate the prevalence of depression and post-traumatic stress among parents of children with cancer.

**Methods:**

Our study was cross-sectional over a period of 1 month in the medical carcinology department at the Salah Azaeiz Institute. We used the Beck Depression Scale II and the Post Traumatic Stress Disorder Cheklist-Civilian assessements.

**Results:**

Thirty parents participated in our study. Most of whom were mothers (73%). The educational level was primary in 63% of cases and socio-economic level was average in 60% of parents. 40% of parents were assessed within six months after diagnosis. Prevalence of depressive disorder and post-traumatic stress disorder were 73% and 57% respectively. The low educational level was correlated to the presence of these two disorders. Similarly, the low educational level was correlated to the presence of depressive disorders (p=0.008). The number of children in the family was also associated to the presence of post-traumatic stress disorder (p=0.029).

**Conclusions:**

The prevalence of depressive and post-traumatic stress disorders was high among parents of children diagnosed with cancer. The low socio-economic and educational level and the large number of children in the family are risk factors for psychological distress. Psychosocial support should be offered to parents of children with cancer to optimize the management of this disease.

**Disclosure:**

No significant relationships.

